# IL20RA Is the Key Factor Contributing to the Stronger Antioxidant Capacity of Rongchang Pig Sertoli Cells

**DOI:** 10.3390/antiox13121545

**Published:** 2024-12-17

**Authors:** Qi-Yue Zheng, Li-Fei Xiao, Tian-Yi An, Liang Zhang, Xi Long, Qing Wang, Xian-Zhong Wang, Hong-Mei Pan

**Affiliations:** 1 Chongqing Academy of Animal Science, Chongqing 402460, China; 2 Chongqing Key Laboratory of Forage and Herbivore, College of Veterinary Medicine, Southwest University, Chongqing 400715, China

**Keywords:** sertoli cells, oxidative stress, pig breeds, LPS, IL20RA

## Abstract

Variations in disease resistance among pig breeds have been extensively documented, with Sertoli cells (SCs) playing a pivotal role in spermatogenesis. Infections can induce oxidative stress, which can lead to damage to these cells. This study aimed to compare the levels of oxidative stress in SCs from Rongchang and Landrace pig breeds following LPS challenge. SCs were isolated, cultured, and stimulated with LPS to assess cell viability and markers of oxidative stress. Cell viability was evaluated along with oxidative stress markers such as reactive oxygen species (ROS), mitochondrial superoxide, malondialdehyde, and antioxidant enzymes. Mitochondrial function was assessed using JC-1 and Calcein AM probes. Transcriptomic analysis identified differentially expressed genes (DEGs), while ingenuity pathway analysis (IPA) explored enriched pathways. IL20RA, identified through transcriptomics, was validated using the siRNA knockdown technique. The results showed that Rongchang SCs exhibited lower levels of oxidative stress compared to Landrace SCs along with higher activity of antioxidant enzymes. IL20RA emerged as a key regulator since its knockdown affected mitochondrial superoxide production and catalase secretion. The findings suggest that Rongchang SCs possess superior antioxidant capacity, possibly due to the IL20RA-mediated protection of mitochondria, thereby providing insights into breed-specific resistance against oxidative stress and highlighting the role of IL20RA in maintaining stem cell function.

## 1. Introduction

Different pig breeds exhibit varying levels of disease resistance, which can have profound implications for the health, growth, and reproductive performance of these animals. This genetic diversity in disease susceptibility is crucial for the management and productivity of swine populations. For example, during an epidemic outbreak on a pig farm, the growth and reproductive performance of Landrace pigs can be significantly impaired, while Min pigs, native to China, often remain unaffected even when housed under the same conditions [1]. Therefore, it is essential to investigate the reasons behind the disease resistance found in diverse pig breeds.

This differential resistance is further highlighted by studies that have compared the immune responses and disease outcomes in different pig breeds. Proteomic and transcriptomic analyses have identified 13 quantitative trait loci associated with immune responses to diseases [2]. In a comparative study analyzing gene expression profiles in lung tissue samples, Dapu Lian pigs, an indigenous Chinese breed, were found to have a lower proportion of CD4+ cells and a reduced CD4+/CD8+ ratio compared to crossbred pigs. Additionally, crossbred pigs exhibited significantly higher levels of the cytokines IL-10 and TNF-α than Dapu Lian pigs [3]. Understanding the genetic and immunological foundations of disease resistance is crucial for advancing and refining pig breeding programs.

Sertoli cells (SCs) are the exclusive somatic cells within the seminiferous tubules and play a pivotal role in maintaining testicular immunity [4]. They ensure the smooth progression of spermatogenesis and protect germ cells from immune cell attacks by establishing a unique immune-privileged status [5]. Initially, SCs safeguard germ cells from immunological destruction through the blood–testis barrier (BTB) [6]. Simultaneously, they express various immunomodulatory factors such as Transforming Growth Factor-beta (TGF-β), Indoleamine 2,3-dioxygenase (IDO), Galectin-1, and Activin A. These factors induce the formation of regulatory T cells (Tregs) and tolerogenic dendritic cells (DCs), creating a local immune tolerance environment that shields germ cells from autoantigen attacks [7,8]. Moreover, Sertoli cells inhibit NK, B, and T cell proliferation while expressing multiple complement inhibitory factors to prevent complement-mediated cytolysis [9,10,11,12]. In response to inflammation stimulated by TGF-β, Sertoli cells express Galectin-1, which exerts pro-inflammatory effects through the Mitogen-Activated Protein Kinase (MAPK) signaling pathway [12]. Additionally, they express the serine protease inhibitor (SERPIN) G1 to counteract the amplification of the MAPK cascade. The balance between these two opposing effects determines cellular fate [7]. Therefore, it is crucial for SCs to maintain homeostasis in order to support immune privilege and overall health of seminiferous tubules.

Oxidative stress refers to an imbalance between the generation of oxidative free radicals in response to adverse stimuli and the body’s antioxidant defenses, resulting in oxidative damage to tissues and cells [13]. Infections lead to the excessive production of reactive oxygen species (ROS), causing cellular damage and abnormalities in sperm motility [14,15]. *Escherichia coli*, a Gram-negative bacterium, can induce ROS generation and oxidative stress in the testes [16]. The induction of oxidative stress led to elevated levels of mitochondrial ROS generation, lipid peroxidation, and DNA double-strand breaks in the TM4 Sertoli cell line compared to the vehicle control [17]; it also disrupted blood–testis barrier (BTB) function and caused the loss of BTB-associated proteins including ZO-1, N-cadherin, Occludin, F11R, Claudin-11, Connexin-43, and F-actin [18,19]. These findings demonstrate that when homeostasis within Sertoli cells is compromised under these conditions, Sertoli cells undergo apoptosis, leading to a reduced sperm count and abnormalities in sperm morphology and motility [20,21,22].

Lipopolysaccharide (LPS) is widely acknowledged as a standard model for investigating inflammatory and infectious responses both in vitro and in vivo [23]. Even at low doses, systemically administered LPS can cause significant testicular damage, characterized by pronounced signs of inflammation and germ cell impairment [24]. LPS has the ability to induce the release of pro-inflammatory cytokines such as tumor necrosis factor-alpha (TNF-α), interleukin-1 (IL-1), and interleukin-6 (IL-6). The acute-phase response disrupts spermatogenesis entirely by activating NF-κB, promoting support cell death and autophagy, thereby impairing male reproductive function in vitro [25]. The LPS stimulation of Sertoli cells can disrupt the intracellular antioxidant balance, triggering ROS production and causing oxidative stress while also reducing glutathione reductase and superoxide dismutase activities [26]. However, it remains unclear whether there are differential responses to LPS stimulation between Sertoli cells from Landrace and Rongchang pigs.

The objective of this study is to investigate the response characteristics of SCs from different pig breeds under LPS stimulation, focusing on elucidating the interbreed variations in antioxidant capabilities and oxidative stress resistance among SCs.

## 2. Materials and Methods

### 2.1. The Isolation, Culture, and Identification of SCs

The animal experiments were conducted strictly in accordance with the Chinese National Guidelines for Laboratory Animal Care and Use, under the supervision and approval of the Institutional Animal Care and Use Committee of Southwest University, Chongqing, China (SWU LAC-2022100214). Testes from two different pig breeds were obtained from the breeding farm of the Chongqing Academy of Animal Sciences in Rongchang District, Chongqing, China. Testis samples were collected from 21-day-old boars of both breeds under aseptic conditions, following the same sampling procedure. Pigs were raised in identical nutritional and environmental conditions [27]. Specifically, cells were cultured in Dulbecco’s Modified Eagle Medium (DMEM)/F12 supplemented with 10% Fetal Bovine Serum (Gibco, New York, NY, USA). The isolation and culture methods for the Sertoli cells of both Landrace and Rongchang breeds were consistent with our previous publications [28]. Identification of Sertoli cells was performed after 3 days of culture at 32 °C with 5% CO_2_ for Landrace breed, while for Rongchang breed it was done after 4 days once similar cell morphology and size were observed. The purity of SCs was assessed using immunofluorescence with GATA-binding protein 4 (GATA-4) as a specific marker. After rinsing with phosphate-buffered saline (PBS), the cells were fixed with 4% paraformaldehyde (PFA) and permeabilized with 1% Triton X-100. Subsequently, each well was treated with 500 μL of fluorescently labeled secondary antibody Rabbit Anti-GATA-4 (1:500, Bioss, Beijing, China) and incubated in the dark at room temperature for 2 h within a light-shielded box. Afterward, nuclear staining was performed by adding 200 μL of DAPI (1:200, Beyotime, Shanghai, China) at room temperature in the dark for 3 to 5 min. Finally, cells were observed under a fluorescence microscope (DM2500; Leica, Wetzlar, Germany). Only cells exhibiting purity level equal to or higher than 95% were utilized [29].

### 2.2. Cell Viability Assay

The cell proliferation assay was performed using a CCK-8 kit (Biosharp, Hefei, China) based on the WST-8 method. Initially, 3000 cells were seeded into each well of multiple 96-well plates. Subsequently, varying concentrations of LPS (0, 5, 10, 30, 50, 100, and 200 µg/mL) were added to the wells. The plates were categorized into groups corresponding to different time points: 0, 2, 4, 8, 12 and 24 h. Blank controls and drug blank groups were included in the experimental design. To each well containing culture medium (200 µL), a solution of CCK-8 (20 µL) was added. After incubation for two hours at room temperature, the absorbance at a wavelength of 450 nm was measured using a microplate reader from Bio-Rad (Hercules, CA, USA) [30].

### 2.3. Detection of Intracellular ROS and MSR

The 2′,7′-dichlorofluorescin diacetate (DCFH-DA) assay kit (Elabscience Biotechnology Co., Ltd., Wuhan, China) was utilized for quantifying the production of ROS within cells [31]. Sertoli cells were incubated with DCFH-DA at 37 °C in a light-free environment for 20 min, followed by three washes with serum-free medium (Gibco). The fluorescence intensity of DCFH-DA was measured using a fluorescence microplate reader (Molecular Devices, Shanghai, China) at excitation/emission wavelengths of 500 nm/525 nm. The data regarding DCFH-DA fluorescence were collected using the SoftMax Pro version 7.0 software from Molecular Devices. To detect mitochondrial superoxide generation, the MitoSOX Red (MSR) assay from Thermo Fisher (Waltham, MA, USA) was used as a live-cell fluorescent probe specifically targeting mitochondria [32]. The MSR reagent stock solution was prepared by dissolving it in 13 μL of anhydrous DMSO to obtain a 5 mM solution. A volume of 5 μL from this stock solution was added to 50 mL of HBSS (Hank’s Balanced Salt Solution) containing calcium and magnesium (Gibco), resulting in a working solution concentration of 500 nM. One milliliter of the MSR working solution was applied to cells in each well of a six-well plate. Subsequently, the cells were incubated at 37 °C in a 5% CO_2_ atmosphere in the dark for up to 30 min. Following incubation, the cells were gently washed three times with warm HBSS with calcium and magnesium or an appropriate buffer at 37 °C. The fluorescence of MSR was measured within 2 h at excitation/emission wavelengths of 500 nm/525 nm.

### 2.4. Biochemical Analysis

The SCs were collected using PBS and disrupted with an ultrasonic homogenizer (Scientz, Ningbo, China). After centrifugation at 4 °C and 12,000× *g* for 10 min, the supernatant was obtained, and the protein concentration was determined using a BCA protein assay kit (Beyotime Biotech, Shanghai, China). Various detection kits (Jiancheng, Nanjing, China) were utilized as per their respective protocols: the Microscale Malondialdehyde (MDA) assay kit employed the TBA method for detection [33], the Reduced Glutathione (GSH) assay kit used the spectrophotometric method [34], and the Catalase (CAT) assay kit employed the ammonium molybdate method [35]. Additionally, the water-soluble tetrazolium salt (WST)-1 method was utilized to evaluate superoxide dismutase (SOD) [36]. The products of these reactions were detected at different wavelengths (562 nm, 532 nm, 420 nm, 450 nm, 405 nm), as specified by the respective kit instructions.

### 2.5. Assessment of Mitochondrial Function

The Mitochondrial Permeability Transition Pore (MPTP) assay kit from Beyotime employs a hydrophobic dye, Calcein AM, which selectively stains viable cells to assess the extent of mitochondrial permeability transition pore opening [37]. Following trypsin digestion for 3 min, treated cells were resuspended in culture medium and centrifuged at room temperature for 5 min at 1000× *g*, discarding the supernatant afterward. Subsequently, the cells were stained with a solution containing Calcein AM and a fluorescence quenching agent, followed by incubation in the dark at 37 °C for 30 min. After incubation, samples were placed on ice, gently mixed, and analyzed using an Agilent flow cytometer with excitation and emission wavelengths set to 494 nm and 517 nm, respectively. Additionally, samples were excited with a 488 nm laser and imaged using a fluorescence microscope equipped with a 494/517 nm filter set.

The Mitochondrial Membrane Potential Assay Kit with JC-1 from Beyotime employs JC-1 as a fluorescent probe for rapid detection of mitochondrial membrane potential (MMP, ΔΨm) [38]. Cells were washed with PBS, and then the appropriate volume of JC-1 staining working solution was added, mixed thoroughly, and incubated at 37 °C in a cell culture incubator for 20 min. The samples were visualized using a fluorescence microscope. The fluorescence of JC-1 monomers was measured at excitation/emission wavelengths of 490 nm/530 nm, while the fluorescence of JC-1 aggregates was measured at 525 nm/590 nm.

### 2.6. RNA Sequencing Data Analysis

The primary Sertoli cells were collected from each breed, resulting in a total of 4 groups for transcriptome analysis. Each group had three replicates under both control and treatment conditions, leading to a total of 12 samples. LC-Bio Technology in Hangzhou, China, managed the workflow, including RNA isolation and RNA-seq analysis.

Total RNA was meticulously extracted from the samples using Trizol (Takara, Shiga, Japan) reagent and evaluated for both purity and quantity using a Bioanalyzer 2100 (Agilent, Santa Clara, CA, USA). Only RNA exhibiting high integrity, as indicated by an RNA Integrity Number (RIN) greater than 7.0, was chosen for library construction. Subsequently, mRNA was purified and fragmented under stringent conditions before reverse transcription into cDNA using SuperScript™ II Reverse Transcriptase (Invitrogen, Waltham, MA, USA). Finally, the cDNA was processed for indexed adapter ligation.

After ligation, the cDNA libraries were subjected to UDG enzyme (NEB, Ipswich, MA, USA) and amplified through PCR to achieve an average insert size of 300 ± 50 bp. Subsequently, these libraries were sequenced on an Illumina NovaSeq™ 6000 platform (LC-Bio Technology, Hangzhou, China), resulting in a substantial yield of 2 × 150 bp paired-end reads.

The data processing commenced by employing Cutadapt for rigorous filtration, eliminating adapter contaminants, polyA/polyG sequences, unknown nucleotides, and low-quality bases [39]. The quality of the resultant clean reads was assessed using FastQC [40], ensuring the removal of reads that contained adapter contamination, low-quality bases, and undetermined bases using default parameters. Subsequently, the clean reads were aligned to the reference pig genome (Sscrofa11.1) utilizing HISat2 version 2.2.1 [41], allowing for multiple alignments with stringent mismatch criteria.

The alignment data facilitated the construction of a comprehensive transcriptome through String Tie version 2.1.6 and gffcompare version 0.9.8, enabling the quantification of gene abundance via FPKM values [41,42,43]. Differential Expression Gene (DEG) Analysis: Gene expression analysis was conducted using DESeq version 2 software to compare two different groups, and edgeR (Bioconductor—edgeR, https://bioconductor.org/packages/release/bioc/html/edgeR.html, accessed on 1 October 2024) was used for pairwise comparisons [44,45,46]. The genes with the parameter of false discovery rate (FDR) below 0.05 and absolute fold change > 2 were selected. Enrichment analysis of DEGs against the GO and KEGG databases was performed to discern significantly affected biological functions and pathways [47,48,49].

### 2.7. Total RNA Extraction and Reverse Transcription Quantitative PCR (RT-PCR)

Total RNA was extracted from SCs using an RNA extraction kit (Goonie, Guangzhou, China). Adhering to the manufacturer’s instructions for the All-In-One 5× RT MasterMix kit (ABM, Richmond, BC, Canada), the components were mixed and briefly centrifuged. The reverse transcription system consisted of the following components: 4 μL of All-In-One 5× RT MasterMix, 2 μg of total RNA or poly(A)+ mRNA per reaction, and nuclease-free H_2_O to reach a final volume of 20 μL. The RNA was reverse-transcribed to complementary DNA (cDNA) using a PCR thermal cycler from Bio-Rad. The qRT-PCR reaction mixture was prepared according to the protocol provided with the BlasTaq™ 2× qPCR MasterMix kit (ABM). The reaction components included 5 μL of BlasTaq™ 2× qPCR MasterMix, 0.4 μL of each Forward Primer (10 μM), 0.4 μL of Reverse Primer (10 μM), 2 μL of template DNA, and nuclease-free H_2_O to adjust the final volume to 10 μL. qPCR was performed on a quantitative real-time PCR instrument from Bio-Rad using gene-specific primers. The qPCR protocol commenced with an initial denaturation step at 95 °C for 3 min to activate the DNA polymerase. This was followed by 40 cycles of denaturation at 95 °C for 15 s and annealing/extension at 60 °C for 1 min per cycle. Relative quantification of gene expression was determined using the 2^−ΔΔCt^ method. β-actin was employed as a housekeeping gene for normalization. Specific gene details are listed in Table 1.

### 2.8. Western Blotting

SCs were washed with PBS and then lysed using a lysis buffer containing protease and phosphatase inhibitor cocktails in a radio-immunoprecipitation assay (RIPA) buffer (Thermo Fisher Scientific, Waltham, MA, USA). Protein concentrations were determined using a BCA protein assay kit from Beyotime Biotech. Equal amounts (20 μg) of protein samples from Sertoli cells were loaded onto an SDS-PAGE gel. The gel was heated to 37 °C for 30 min to induce protein denaturation. Electrophoresis was performed at 300 V for 30 min, followed by protein transfer onto a PVDF membrane at 100 V for 100 min. The membrane was then blocked with 5% bovine serum albumin at room temperature for 2 h. The membrane was incubated with primary antibodies, namely IL20RA (1:800, Abclonal, Wuhan, China) and β-actin (1:3000, Thermo Fisher Scientific, Waltham, MA, USA). This was followed by incubation with a secondary antibody: anti-rabbit IgG (1:2000, Beyotime, Shanghai, China). All antibodies were diluted in 1×TBST before use. Protein bands were visualized using a chemiluminescence reagent from Biosharp (China) and detected with the ChemiDocXRS+ imaging system (Bio-Rad, Hercules, CA, USA). The bands were analyzed using ImageJ version 1.51j8 software (NIH, Bethesda, MD, USA) [50].

### 2.9. Small Interfering RNA

Several small interfering RNA (siRNA) sequences targeting IL20RA were designed and synthesized by Wubio in Chongqing, China, with the specific sequences listed in Table 2. Transfection was initiated when the cell density reached approximately 70–80%. Before the transfection, each well of a six-well plate was replenished with 2 mL of fresh culture medium. The siRNAs were prepared as a 20 µM stock solution using nuclease-free water. Transfection was carried out using Lipo8000™ transfection reagent from Beyotime in Shanghai, China, following the manufacturer’s instructions. A ratio of siRNA to Lipo8000 of 25:1 (pmol to µL) was added to the cell culture supernatant and incubated for 10 h [51]. After the medium was replaced, the cells were further cultured for 36 to 60 h. Samples were collected at appropriate time points as dictated by the experimental design.

### 2.10. Statistical Analysis

Statistical analysis was performed utilizing SPSS (V22) software (IBM Corporation, Armonk, NY, USA). Initially, the data were assessed for normality. Non-normally distributed data were subjected to a sine transformation prior to further analysis. Each experiment was independently replicated 3 times, and comparisons between two groups were made using the unpaired Student’s *t*-test to determine statistical significance. For experiments with three or more groups, one-way ANOVA was used, followed by Tukey’s post hoc test for multiple comparisons to evaluate differences between groups. Inter-porcine breed comparisons and the assessments of time and concentration in the CCK-8 screening were conducted using two-way ANOVA, with Sidak’s multiple comparison test to ascertain significance among multiple groups. Data visualization was carried out with GraphPad Prism version 10.1.2, and all data are expressed as the mean ± SEM. * *p* < 0.05, significant; ** *p* < 0.01, highly significant; *** *p* < 0.001, very significant.

## 3. Results

### 3.1. Identification of the Purity of SCs from Landrace and Rongchang Pigs

**Figure 1:**
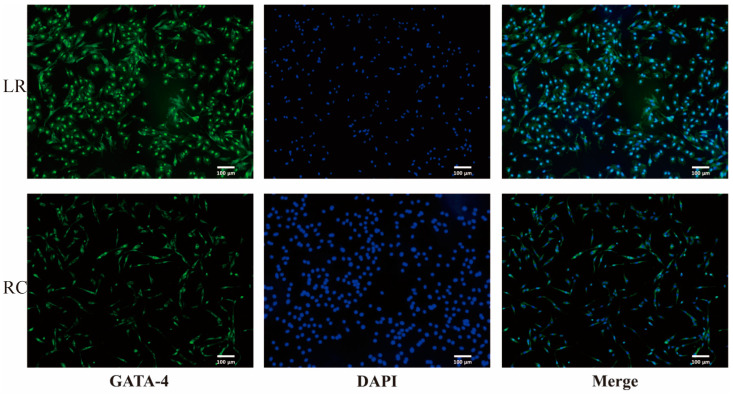
Purity identification of SCs cultured in vitro. Green fluorescence represents GATA-4, and blue fluorescence represents DAPI. Scale bar = 100 μm. SCs, Sertoli cells.

The SCs isolated from two pig breeds both exhibited a rounded morphology. However, differences in morphological features were observed. Specifically, under normal cellular functional conditions, SCs from Rongchang pigs appeared more elongated and smaller compared to those from Landrace pigs. For subsequent experiments, a three-day culture period following recovery is necessary for Landrace SCs. In contrast, Rongchang pig SCs are expected to achieve cell density and purity comparable to those of Landrace pigs by the fourth day of cultivation, which is designated as the time point for purity verification. Using GATA-4 as a molecular marker for immunofluorescence identification, we observed that the purity of Landrace SCs was 95.61%, while Rongchang pig SCs had slightly higher purity at 96.62% (Figure 1).

### 3.2. Cell Viability Between Two SCs Stimulated by LPS

**Figure 2:**
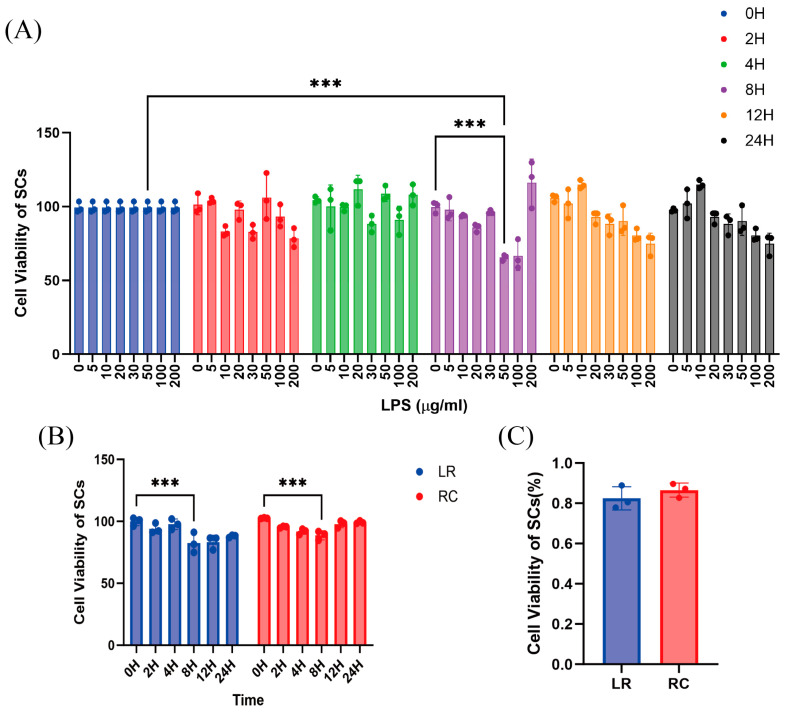
Differences in cell viability between two SCs stimulated by LPS. (**A**) Cellular viability of Landrace pig SCs treated with LPS at various times and concentrations. (**B**) Cellular viability of Landrace and Rongchang pig SCs treated with 50 µg/mL LPS. (**C**) Cellular viability normalized to the control group after treatment with 50 µg/mL LPS for 8 h. *** *p* < 0.001, even more significant. LPS, lipopolysaccharide.

The optimal dose and duration of LPS stimulation were determined by employing SCs from Landrace pigs. Different concentrations of LPS were applied for varying durations, and cell viability was assessed using the CCK-8 assay. The results demonstrated a significant decrease in cell viability after treating cells with 50 µg/mL LPS for 8 h (Figure 2A). Subsequently, the viability of SCs from both pig breeds was evaluated under the same concentration of 50 µg/mL LPS but with varying exposure times. The findings revealed a significant reduction in viability for both breeds at the 8 h time point (Figure 2B). Normalization to the control group (0 h) at the 8 h time point showed no significant differences in cell viability between the two breeds (Figure 2C). Consequently, 8 h exposure to 50 µg/mL LPS was chosen as the oxidative stress model for this experiment.

### 3.3. Differences in Oxidative Stress Status of SCs Before and After LPS Treatment in Two Breeds

**Figure 3:**
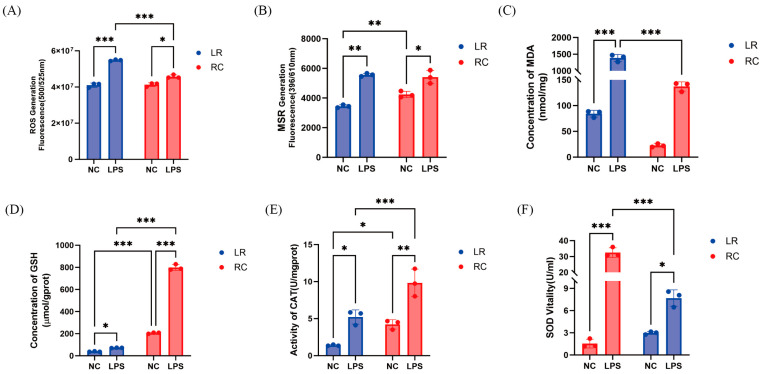
Differences in oxidative stress status of SCs before and after LPS treatment in two breeds. (**A**–**C**) Comparative assessment of oxidative stress levels and antioxidant enzyme activities in SCs from two pig breeds under identical treatment conditions. Intracellular levels of ROS, MSR, and MDA were measured in SCs from both pig breeds following exposure to the same concentration of LPS. The content of GSH (**D**), CAT activity (**E**), and SOD content (**F**) were evaluated under LPS treatment. * *p* < 0.05, significant; ** *p* < 0.01, highly significant; *** *p* < 0.001, even more significant. ROS, reactive oxygen species; MSR, MitoSOX Red; MDA, malondialdehyde; GSH, glutathione; CAT, catalase; SOD, superoxide dismutase.

To investigate breed-specific differences in ROS generation following LPS exposure, we quantified levels of ROS, MSR, and MDA in SCs from two pig breeds. Initially, both breeds exhibited similar levels of ROS before LPS treatment. However, after treatment, there was a significant increase in ROS levels. Notably, SCs from Rongchang pigs showed a comparatively lower increase than those from Landrace pigs (Figure 3A). To measure MSR levels specifically related to mitochondrial superoxide production, we employed the fluorescent probe MitoSOX Red. Our results revealed that Rongchang pig SCs displayed a smaller increase in MSR post-LPS treatment compared to Landrace SCs (Figure 3B). The trends observed with ROS and MSR were mirrored by MDA content; Rongchang pig SCs exhibited significantly lower MDA levels after LPS treatment (Figure 3C), indicating differences in oxidative stress response between the breeds under these conditions. Furthermore, we evaluated antioxidant enzyme activity by measuring GSH, CAT, and SOD levels. Prior to LPS treatment, Rongchang pig SCs demonstrated higher GSH and CAT levels but lower SOD levels compared to Landrace SCs (Figure 3D–F). Following LPS treatment, both breeds experienced the significant upregulation of GSH, CAT, and SOD; however, the increase was more pronounced in Rongchang pig SCs. These findings suggest that Landrace SCs exhibit a more pronounced oxidative stress response following LPS treatment compared to Rongchang pig SCs. Conversely, the higher antioxidant enzyme activity in Rongchang pig SCs suggests a stronger antioxidant capacity.

### 3.4. Differences in Oxidative Stress Are Associated with Mitochondrial Damage

**Figure 4:**
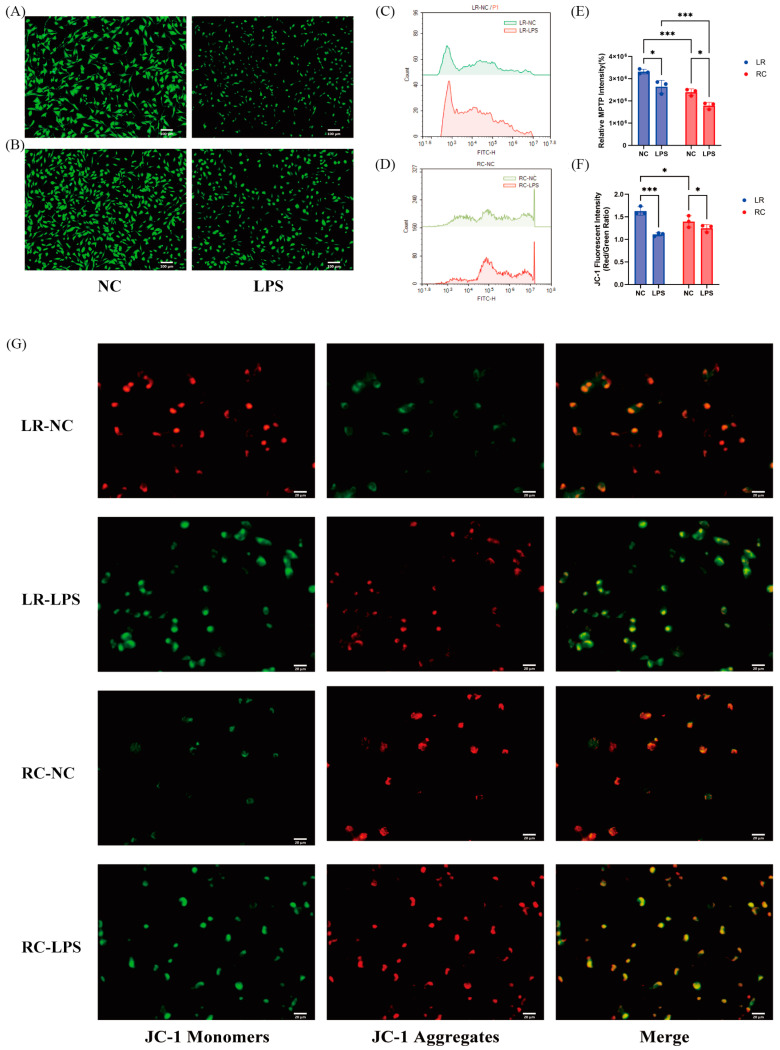
Differences in oxidative stress are associated with mitochondrial damage. (**A**,**B**) Changes in MPTP fluorescence before and after treatment in SCs from Landrace (**A**) and Rongchang pigs (**B**), green fluorescence represents Calcein AM (scale bar = 100 μm). (**C**,**D**) Flow cytometry assessment of MPTP opening in SCs from Landrace (**C**) and Rongchang pigs (**D**). (**E**) Bar graph representing the MPTP opening rate in both types of SCs. (**F**) The impact of both SCs on mitochondrial membrane potential (MMP). Red fluorescence indicates high MMP; green fluorescence indicates low MMP. A lower red-to-green ratio indicates higher MMP. (**G**) MMP in both types of SCs observed under a fluorescence microscope, green fluorescence represents JC-1 monomers and red fluorescence represents JC-1 aggregates (scale bar = 20 μm). * *p* < 0.05, significant; *** *p* < 0.001, even more significant. MPTP, mitochondrial permeability transition pore.

The mitochondrial permeability transition pore (MPTP), a non-selective channel located in the inner mitochondrial membrane, triggers a cascade of cell death reactions upon opening. In this study, Calcein AM, a specific fluorescent probe, was used to detect MPTP opening. Flow cytometry and fluorescence microscopy observations revealed that Landrace SCs exhibited a greater reduction in fluorescence intensity, indicating a higher degree of MPTP opening compared to Rongchang SCs (Figure 4A–F). We also evaluated MMP (ΔΨm) using a JC-1 fluorescent probe. A decrease in MMP signifies mitochondrial dysfunction and is an early indicator of apoptotic events. Figure 4G findings suggest that after LPS treatment, the reduction in red fluorescence (associated with JC-1 monomer) and increase in green fluorescence (associated with JC-1 polymer) were more pronounced in Landrace pigs than in Rongchang pigs. These results indicate differences exist between the two breeds of SCs regarding mitochondrial function following LPS treatment.

### 3.5. Transcriptomics Reveals SC Differences Between Two Breeds

**Figure 5:**
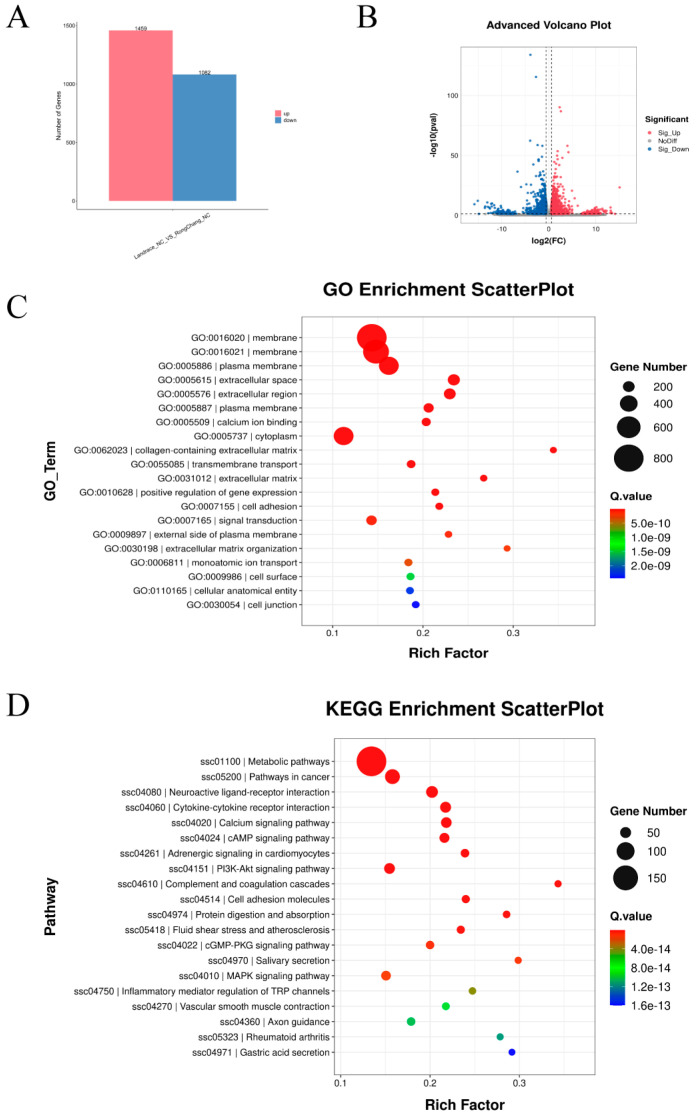
Transcriptomics uncovers differences in SCs between two pig breeds. Bar chart (**A**) and volcano plot (**B**) depicting the number of differentially expressed genes between Landrace and Rongchang SCs. Transcriptomic analysis of GO term (**C**) and KEGG enrichment analysis (**D**) for Landrace and Rongchang SCs.

The comparison between Rongchang SCs and Landrace SCs revealed the significant upregulation of 1459 genes and the downregulation of 1082 genes (Figure 5A,B). Gene ontology (GO) enrichment analysis identified a total of 1259 significantly enriched GO terms (*p* < 0.05), particularly those associated with membrane, plasma membrane, and cytoplasm. These included terms related to membrane composition, repair, and signal transduction response (GO:0016020), the overall structure of the cell membrane (GO:0005886), and the intrinsic components of the cell membrane (GO:0005887) (Figure 5C). Furthermore, KEGG enrichment analysis revealed that a total of 254 pathways were significantly enriched (*p* < 0.05), especially those involved in metabolism, cancer pathways, cytokine interactions, and the PI3K-Akt signaling pathway. These included metabolic pathways (ssc01100), pathways in cancer (ssc05200), cytokine–receptor interaction (ssc04060), and the PI3K-Akt signaling pathway (ssc04151) (Figure 5D). The transcriptional results indicate inherent differences in cellular membranes and membrane-associated proteins between the two breeds. Additionally, they highlight disparities in genes related to metabolism and other biological processes prior to treatment.

### 3.6. Transcriptome Characterization of the SCs Response to LPS in Two Breeds

**Figure 6:**
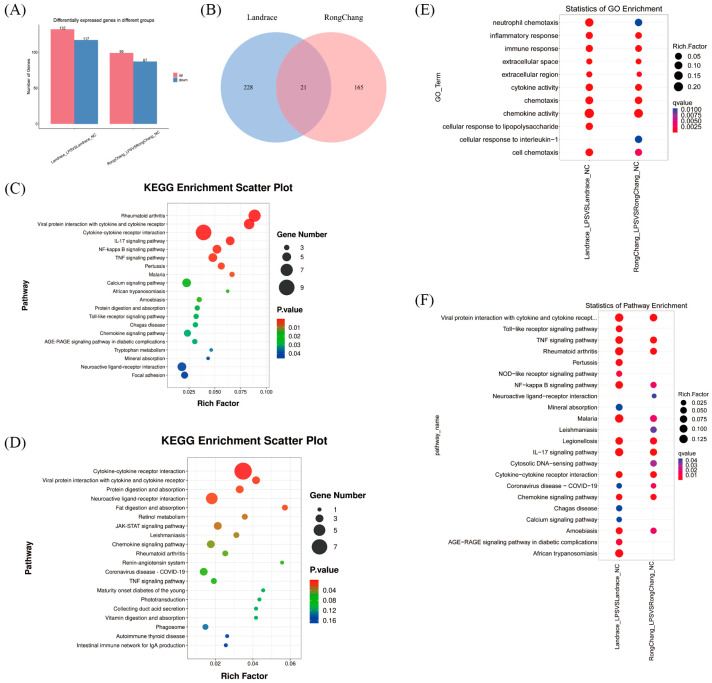
Transcriptome characterization of the SCs response to LPS in two breeds. (**A**) Bar chart of differential gene count in SCs from two pig breeds following treatment. (**B**) Venn diagram analysis of all differential genes in SCs from the two pig breeds (red represents Landrace, blue represents Rongchang). (**C**) KEGG enrichment analysis of upregulated genes following treatment in Landrace pigs. (**D**) KEGG enrichment analysis of upregulated genes following treatment in Rongchang pigs. (**E**) GO term enrichment analysis of non-intersecting upregulated genes from the Venn diagram analysis of all differential genes following treatment in SCs from the two pig breeds. (**F**) KEGG enrichment analysis of the same non-intersecting upregulated genes.

Comparative transcriptomic analysis following LPS treatment revealed distinct responses between Landrace and Rongchang SCs. In Landrace pigs, LPS treatment resulted in the upregulation of 132 genes and the downregulation of 117 genes compared to the control group; Rongchang pigs exhibited the upregulation of 99 genes and the downregulation of 87 genes (Figure 6A). KEGG enrichment analysis post-LPS treatment in Landrace pigs highlighted inflammatory signaling pathways, including rheumatoid arthritis, cytokine–receptor interaction, IL-17, NF-κB, and TNF signaling pathways (Figure 6C). In contrast, Rongchang pigs showed enrichment in cytokine–receptor interaction and the JAK-STAT signaling pathway (Figure 6D).

The Venn diagram analysis revealed 21 intersecting differentially expressed genes between the two breeds, with 228 unique to Landrace pigs and 165 unique to Rongchang pigs (Figure 6B). GO term enrichment analysis of the unique genes indicated that Landrace pigs exhibited an enriched response to lipopolysaccharide, which was not observed in Rongchang pigs; conversely, Rongchang pigs showed an enriched cellular response to IL-1, which this was not observed in Landrace pigs (Figure 6E).

KEGG enrichment analysis revealed that the Toll-like receptor signaling pathway and Nod-like receptor signaling pathway were significantly enriched in Landrace pigs, whereas Rongchang pigs did not show this enrichment (Figure 6F). These results suggest that under identical LPS stimulation conditions, Landrace pigs exhibit a more robust inflammatory response than Rongchang pigs, potentially explaining the previously reported differences in oxidative stress between these two breeds.

### 3.7. Identification of Molecular Gene Expression Signatures Associated with LPS Treatment of Two Pigs

**Figure 7:**
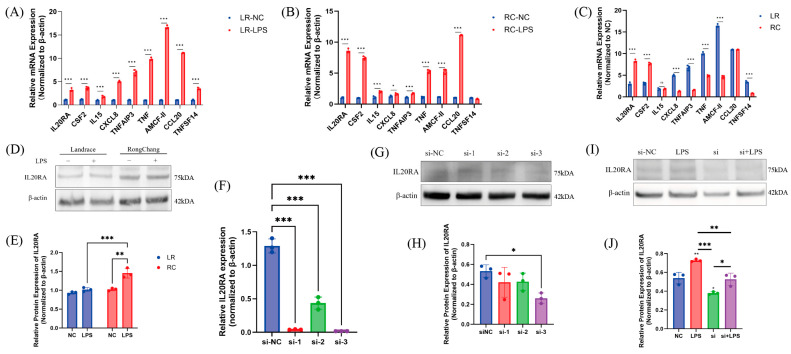
Identification of molecular gene expression signatures associated with LPS treatment of two pigs. Validation of upregulated genes identified by transcriptomics using qPCR, (**A**) Landrace, (**B**) Rongchang. (**C**) Normalization of gene expression differences at the genus between the two types of pigs following their respective treatments compared to control groups. (**D**) Impact of LPS treatment under the same conditions on IL20RA protein levels in SCs of both pig breeds, with β-actin serving as an internal control, and (**E**) presents the quantitative results. (**F**) The effect of different small interfering RNA treatments on IL20RA gene levels in Rongchang SCs; (**G**) shows the impact at the protein level with β-actin as an internal control, and (**H**) the quantitative outcomes. (**I**) IL20RA protein expression levels in Rongchang SCs under interference and LPS treatment, with β-actin as an internal control, and (**J**) the quantitative results. * *p* < 0.05, significant; ** *p* < 0.01, highly significant; *** *p* < 0.001, even more significant. ns: non significant. Quantification of immunoblots was performed using Image Lab 2.0.1 software. An asterisk without an underline indicates statistical significance compared to the respective NC group.

Among the differentially expressed genes, IL20RA, a membrane protein and an upstream regulator of the JAK-STAT pathway, was notably enriched in Rongchang pig SCs. To validate the transcriptomic findings, we performed qPCR on genes that were uniquely upregulated and enriched in specific populations as determined by a Venn diagram comparison. This analysis identified IL20RA, CMF2, IL15, CXCL8, TBFAIP3, TNF, AMCF-2, CXCL20, and TNFSF14 as genes of significant interest. Among these genes, IL20RA and CMF2 exhibited elevated RNA levels in Rongchang SCs compared to Landrace SCs; however, IL15 RNA levels were equivalent across breeds (Figure 7A–C). In contrast, the RNA levels of the other identified genes were higher in Landrace SCs (Figure 7A–C). Further investigation into the protein expression of IL20RA in both types of SCs after LPS treatment revealed a marked increase in Rongchang SCs (Figure 7D,E). To elucidate the functional impact of IL20RA, we employed qPCR and Western blotting to assess the effect of LPS on its expression. Protein expression significantly increased following LPS treatment but was substantially diminished upon siRNA interference (Figure 7F–J); nevertheless, the post-treatment levels still exceeded those in the interference group. These outcomes suggest that IL20RA may play a pivotal role in explaining the divergent inflammatory response tolerance observed between these two pig breeds.

### 3.8. IL20RA Is Identified as the Key Factor Contributing to the Enhanced Antioxidant Capacity of Rongchang SCs

**Figure 8:**
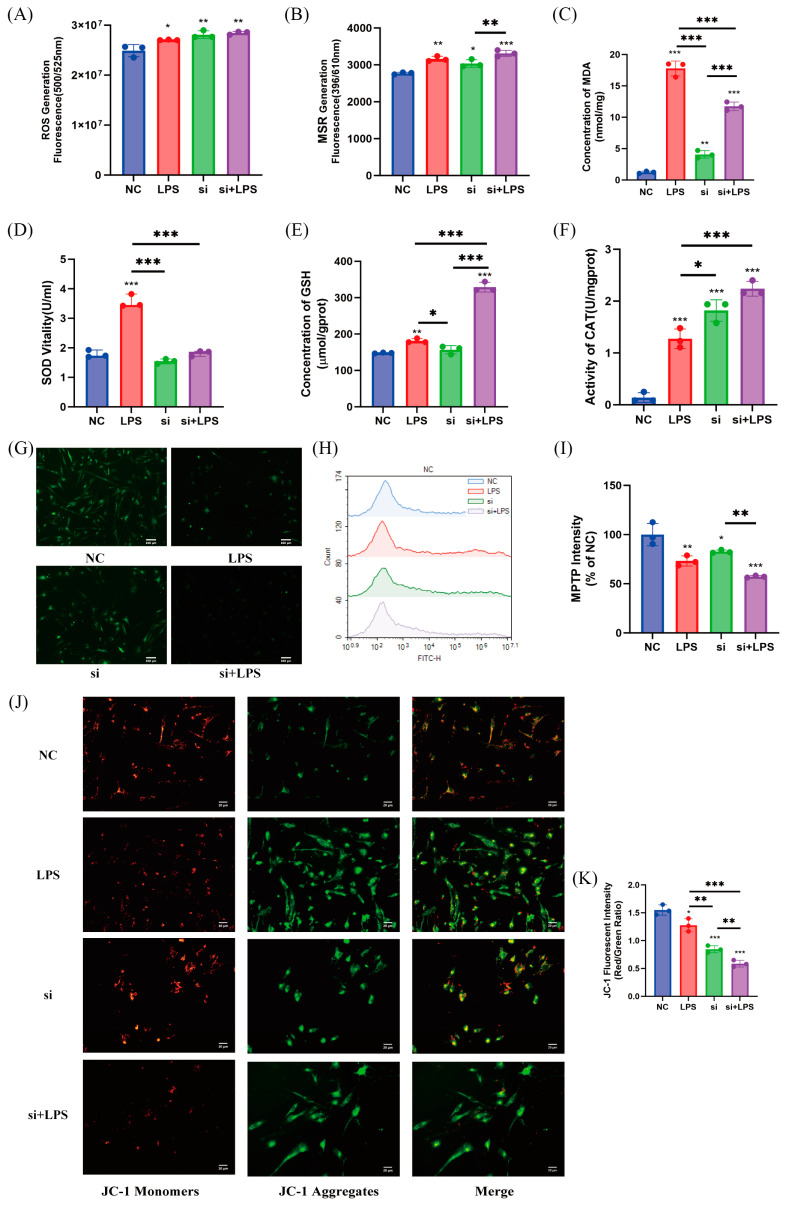
Validation of the oxidative stress effect of LPS on Rongchang SCs under the condition of siRNA interference of IL20RA. The impact of IL20RA knockdown on oxidative stress levels in LPS-treated Rongchang SCs, with (**A**) ROS, (**B**) MSR, and (**C**) MDA assessed. Effects of IL20RA knockdown on the levels of antioxidant enzymes in LPS-treated Rongchang SCs, including (**D**) SOD, (**E**) GSH, and (**F**) CAT. The influence of IL20RA knockdown on the opening degree of MPTP in LPS-treated Rongchang SCs, green fluorescence represents Calcein AM, observed by (**G**) fluorescence microscopy (scale bar = 100 μm) and (**H**) flow cytometry, with (**I**) quantitative statistics from flow cytometry. The effect of IL20RA knockdown on MMP in LPS-treated Rongchang SCs, green fluorescence represents JC-1 monomers and red fluorescence represents JC-1 aggregates, observed by (**J**) fluorescence microscopy (scale bar = 20 μm) and (**K**) fluorescence plate reader for the ratio of red to green fluorescence intensity. * *p* < 0.05, significant; ** *p* < 0.01, highly significant; *** *p* < 0.001, even more significant. An asterisk without an underline indicates statistical significance compared to the respective NC group.

To investigate the regulatory role of IL20RA in the oxidative stress response of Rongchang SCs, an interference study was conducted. Our findings indicate that interference with IL20RA did not significantly affect the production of ROS (Figure 8A). However, interfered cells exhibited higher levels of MSR compared to control cells, with a further increase observed when combined with LPS treatment (Figure 8B). Moreover, the interference + LPS group showed notably lower MDA content than the LPS-only group (Figure 8C). The assessment of antioxidant enzyme levels revealed no significant impact on SOD activity or GSH content upon interfering with IL20RA in Rongchang SCs (Figure 8D,E). In contrast, CAT activity was significantly elevated in the interference group compared to the control group and further increased with LPS treatment relative to the LPS group (Figure 8F).

The impact of IL20RA interference on mitochondrial function was assessed using flow cytometry and fluorescence assays. The results demonstrated that IL20RA interference alone increased the opening degree of MPTP (Figure 7G,H). Furthermore, the fluorescence intensity in the interference + LPS group was significantly reduced compared to the interference group, indicating a greater degree of MPTP opening (Figure 8I). These findings suggest that interfering with the IL20RA gene in Rongchang SCs affects mitochondrial function and subsequently impacts the generation of mitochondrial superoxides.

## 4. Discussion

It is essential to comprehend the variations in disease resistance among species for effective breeding practices. In this study, we conducted a comparative analysis of the oxidative stress response in SCs from the Rongchang and Landrace pig breeds following LPS induction. This comparison revealed significant disparities in antioxidant capacities and mitochondrial functions between the cells of these two distinct pig breeds. The transcriptomic profiles indicated breed-specific enrichment in inflammatory signaling and cellular responses to LPS exposure. Moreover, the knockdown of the IL20RA gene in Rongchang SCs modulated mitochondrial superoxide levels and significantly influenced the secretion of antioxidant enzymes, particularly catalase. These findings suggest that the enhanced antioxidant capacity and reduced mitochondrial damage observed in Rongchang SCs may be attributed to the protective role played by the IL20RA gene. This research has deepened our understanding of variations in antioxidant stress capabilities among different pig breeds while uncovering novel functions of IL20RA in SCs, as well as its role in maintaining cellular homeostasis.

The effects of LPS on cell injury are both cell-specific and breed-specific. In the investigation of cellular viability differences following LPS treatment of the two cell types, the most severe cell damage in Rongchang and Landrace pigs occurred at a concentration of 50 µg/mL after 8 h, which is inconsistent with the response concentrations observed in other cells stimulated by LPS (1 μg/mL in RAW264.7 for 24 h) [52]. This discrepancy may be attributed to the unique immune-privileged characteristics exhibited by SCs [26]. In research inducing inflammation in primary bovine SCs, treatment with 5 μg/mL LPS for 24 h resulted in significant differences compared to the control group, while a concentration of 20 μg/mL showed no difference after 6 h but significant differences after 12 h [53], suggesting that the effects of LPS are species-specific. In time-gradient experiments, at both 12 and 24 h post-stimulation with an identical drug concentration, Rongchang pig cells demonstrated significantly higher viability compared to Landrace pig cells, indicating that Rongchang pigs possess stronger resistance against cellular damage. Not only our experiments but also others have shown variations in immune-privileged characteristics among different pig breeds; for example, Landrace piglets exhibit higher neutrophil values compared to Landrace piglets while showing opposite trends for lymphocyte values [54]. Synthesizing these results, we conclude that Sertoli cells from Rongchang pigs demonstrate stronger resistance against LPS-induced damage, potentially due to their unique immune characteristics.

The disparity between the two breeds arises from variations in antioxidant capacity. When investigating the impact of oxidative stress on cellular damage, elevated levels of ROS can induce mitochondrial dysfunction, consistent with previous research findings [55,56,57,58,59,60]. The functional state of mitochondria directly impacts cell viability as the primary source of intracellular ROS. Our findings demonstrate that Rongchang pigs exhibit lower ROS levels under LPS stimulation. Further investigation was conducted to explore the roles of antioxidant enzymes, including CAT and SOD, in the cellular defense system. These enzymes primarily function in the cytoplasm by decomposing hydrogen peroxide, thereby protecting cells against peroxide-induced toxicity [61]. Experimental results reveal that Rongchang pigs possess a greater capacity to produce CAT, SOD, and GSH following LPS stimulation compared to Landrace pigs, indicating a stronger antioxidant stress response in Rongchang pigs. Moreover, it was observed that mitochondrial damage in Landrace pigs following LPS treatment correlates with oxidative stress levels manifested by higher intracellular ROS and MDA content compared to Rongchang pigs. This result suggests that the disparity in cellular damage under LPS stimulation between these two pig breeds may arise from their distinct responses to oxidative stress.

To elucidate the differential oxidative stress responses between two pig breeds, we conducted a transcriptomic analysis. Prior to treatment, comparative transcriptomic analysis revealed GO enrichment primarily associated with terms related to membranes, including their composition, repair and signal transduction (GO:0016020), overall structure (GO:0005886), and intrinsic components (GO:0005887). These findings suggest significant differences in membrane-associated proteins between the two breeds. Furthermore, TLR4, a pattern recognition receptor present on the cell surface of SCs, can recognize pathogen-associated molecular patterns (PAMPs), such as bacterial LPS. Its activation can initiate a cascade of immune responses, including inflammatory reactions [62]. Zhu et al. reported that the LPS-induced activation of Toll-like receptor and Nod-like receptor signaling pathways [63,64,65] is enriched in Landrace pigs, whereas such enrichment is not observed in Rongchang pigs in our study, suggesting that Rongchang pigs possess a greater tolerance to LPS. Furthermore, Toll-like receptors were not found to be enriched in Rongchang pigs following stimulation. This finding, combined with the previously observed disparities in membrane composition, suggests that variations in membrane receptors between the two pig breeds may contribute to differences in LPS tolerance.

Transcriptomic profiling following LPS treatment in the two Sertoli cell types revealed predominant alterations in pathways associated with inflammation and immunity. KEGG enrichment analysis indicated involvement in cytokine–receptor interaction and the JAK-STAT signaling pathway specifically in Rongchang pigs. Subsequent LPS stimulation of macrophages resulted in an upregulation of inflammatory cytokines, which subsequently activated receptor-associated JAKs, leading to STAT phosphorylation [66,67]. LPS stimulation can also modulate the expression of genes involved in the inflammatory response, including those associated with the NF-κB signaling pathway [23]. This observation aligns with the KEGG changes observed in the transcriptome analysis of Landrace pigs. However, particular attention was given to the JAK-STAT pathway that exhibited enrichment in Rongchang pigs, along with its upstream factor IL20RA, a membrane receptor that showed enrichment upon LPS stimulation in Rongchang pigs as well. The JAK-STAT pathway plays a pivotal role in regulating immune cell development, differentiation, and function, especially in cytokine signaling [68]. It is noteworthy that STAT3 within mitochondria can directly promote Ras-induced transformation and regulate respiration and redox status, indicating that the JAK-STAT signaling pathway not only operates within the nucleus but also actively participates in energy metabolism and determination of cell fate within mitochondria [69].

Subsequently, the differentially upregulated genes identified from the Venn diagram analysis were screened, revealing that IL20RA, CMF2, and IL15 exhibited upregulation in Rongchang pigs, whereas CXCL8, TBFAIP3, TNF, AMCF-2, CXCL20, and TNFSF14 showed upregulation in Landrace pigs. The identification of transcriptional molecules in Landrace pigs demonstrated significant similarities with other studies pertaining to inflammation and oxidative stress [62,70]. While IL20RA has been extensively investigated for its role in immune modulation and autoimmunity, its association with oxidative stress remains relatively unexplored. Previous research has indicated that IL20RA can activate JAK1-STAT3 signaling pathways and is highly expressed in human breast cancer [71]. However, in patients with ovarian cancer, IL20RA is considered a crucial factor for preventing peritoneal metastasis of ovarian cancer cells by potentially creating an immunosuppressive microenvironment that inhibits metastasis [72]. Additionally, the primary focus of research on IL20RA lies within its role in regulating immune responses particularly related to autoimmune diseases such as psoriasis and rheumatoid arthritis [73,74]. Although studies on IL20RA have primarily concentrated on immune modulation and cancer-related aspects, investigations regarding oxidative stress are limited [75]. We have identified IL20RA as a gene with significant differences between two pig breeds from our transcriptome analysis. Given its link to the JAK-STAT pathway, which is not enriched in Large White pigs, we are investigating IL20RA for insights into genetic mechanisms behind breed differences.

To investigate the role of IL20RA in the oxidative stress response of Rongchang Sertoli cells upon LPS stimulation, siRNA targeting IL20RA was employed. Interference with IL20RA demonstrated a more significant regulation of mitochondrial superoxide levels compared to ROS or MDA and also modulated CAT activity. Further examination of IL20RA’s impact on mitochondrial function revealed that its interference increased the opening degree of the MPTP and reduced the mitochondrial membrane potential, indicating that IL20RA regulates mitochondrial function in Rongchang SCs in response to LPS stimulation. We selected a siRNA with high knockdown efficiency for IL20RA, which resulted in low expression levels of the target protein. When introduced into LPS-treated cells, IL20RA levels significantly decreased compared to LPS alone but were not significantly different from the siRNA-only group, likely due to the high silencing efficiency of the siRNA used. This suggests that the minimal difference in IL20RA levels between the siRNA and LPS + siRNA groups could be attributed to the siRNA’s high knockdown efficiency. We believe focusing on IL20RA will help explore the molecular basis of breed differences more effectively. The variation in oxidative stress parameters post-IL20RA knockdown is expected, as these parameters are influenced by multiple factors, but the result showed that IL20RA knockdown has lower antioxidants. STAT3 is localized within mitochondria and participates in cellular respiration by directly regulating complex I of the mitochondrial electron transport chain [76,77]. Transcriptomic analysis also indicated activation of the JAK-STAT pathway upon LPS treatment. Therefore, it can be inferred that IL20RA may influence mitochondrial function through the activation of the JAK-STAT pathway. Differential expression of IL20RA in pig breeds’ SCs may confer protection against mitochondrial damage by reducing oxidative stress. This research emphasizes the involvement of IL20RA in differential tolerance to LPS between the two pig breeds, suggesting that enhanced resistance to LPS observed in Rongchang pigs could be attributed to the activation of IL20RA.

## 5. Conclusions

In summary, this study elucidates the variations in oxidative stress levels and antioxidant capacities in testicular Sertoli cells of two pig breeds under LPS stimulation, as well as the species-specific advantages of the IL20RA gene in indigenous Chinese Rongchang pigs. When subjected to the same LPS challenge, Rongchang pigs demonstrate lower oxidative stress levels and enhanced antioxidant capabilities compared to Landrace pigs. These differences are primarily attributed to the protective role of IL20RA in Rongchang pigs against mitochondrial damage during oxidative stress and its regulatory effect on CAT. These findings contribute to a better understanding of the varying infectious resistance among pig breeds, deepen our comprehension of breed-specific differences in resistance to oxidative stress, and provide novel insights into the role of IL20RA in SC function and survival.

## Figures and Tables

**Table 1:** Sequences of the primers used in this study.

Gene Symbol	GenBank^®^ Accession Number	Forward Primer (5′-3′)	Reverse Primer (5′-3′)
IL20RA	XM_021087511.1	ACACGTCAGGTTTCCCTTTTT	CTGGTGGGTTCCATTGTAGGA
CSF2	NM_214118.2	ATCAAAGAAGCCCTGAGCCTT	GGTTTCATTCATCACAGCCGC
IL15	NM_214390.1	TGCATCCAGTGCTACTTGTGT	CCTGCACTGATACAGCCCAA
CXCL8	NM_213867.1	TGCACTTACTCTTGCCAGAACTG	CAAACTGGCTGTTGCCTTCTT
TNFAIP3	NM_001267890.1	GTGCCCCAGCTTTCTCTCAT	TGGGGGTTTGCTTTGGTTCT
TNF	NM_214022.1	CCAGACCAAGGTCAACCTCC	TTGATCTCGGCACTGAGTCG
AMCF-II	NM_213876.1	AGGCAGAAGTGATAGCCACC	GAGCTTTTGGGTCCAGACAGA
CCL20	NM_001024589.1	GGCTGCTTTGATGTCGGTG	AAGTTGCTTGCTTCTGACTTG
TNFSF14	NM_001260482.1	TGATGCAAGAGCGGAGGC	AGTTTCGTCTCCCACAGCAG
β-actin	AJ312193.1	TCTGGCACCACACCTTCTACAAC	GTCATCTTCTCACGGTTGGCTTTG

**Table 2:** Sequences of siRNA in this study.

Target Gene Gene Accession Number		Forward Primer	Reverse Primer
IL20RA XM_021087511.1	sus-IL20RA-si-1	GAGAUGAUGUCAUGUUCAAUGTT	CAUUGAACAUGACAUCAUCUCTT
	sus-IL20RA-si-2	CAUGAAGAAUAUCCUACAAUGTT	CAUUGAACAUGACAUCAUCUCTT
	sus-IL20RA-si-3	CAGUGUGUGACGAACCAUACGTT	CGUAUGGUUCGUCACACACUGTT
	non-targeting siRNA control (siRNA NC)	UUCUCCGAACGUGUCACGUdTdT	ACGUGACACGUUCGGAGAAdTdT

## Data Availability

The raw sequence data have been submitted to the NCBI Short Read Archive (SRA) with accession number with accession number <PRJNA1172786>. The original contributions presented in the study are included in the article, further inquiries can be directed to the corresponding authors.
